# A case study exploring associations between popular media attention of scientific research and scientific citations

**DOI:** 10.1371/journal.pone.0234912

**Published:** 2020-07-01

**Authors:** P. Sage Anderson, Aubrey R. Odom, Hunter M. Gray, Jordan B. Jones, William F. Christensen, Todd Hollingshead, Joseph G. Hadfield, Alyssa Evans-Pickett, Megan Frost, Christopher Wilson, Lance E. Davidson, Matthew K. Seeley

**Affiliations:** 1 Department of Exercise Sciences, Brigham Young University, Provo, Utah, United States of America; 2 Department of Statistics, Brigham Young University, Provo, Utah, United States of America; 3 University Communications, Brigham Young University, Provo, Utah, United States of America; 4 Harold B. Lee Library, Brigham Young University, Provo, Utah, United States of America; 5 School of Communications, Brigham Young University, Provo, Utah, United States of America; Max Planck Society, GERMANY

## Abstract

The association between mention of scientific research in popular media (e.g., the mainstream media or social media platforms) and scientific impact (e.g., citations) has yet to be fully explored. The purpose of this study was to clarify this relationship, while accounting for some other factors that likely influence scientific impact (e.g., the reputations of the scientists conducting the research and academic journal in which the research was published). To accomplish this purpose, approximately 800 peer-reviewed articles describing original research were evaluated for scientific impact, popular media attention, and reputations of the scientists/authors and publication venue. A structural equation model was produced describing the relationship between non-scientific impact (popular media) and scientific impact (citations), while accounting for author/scientist and journal reputation. The resulting model revealed a strong association between the amount of popular media attention given to a scientific research project and corresponding publication and the number of times that publication is cited in peer-reviewed scientific literature. These results indicate that (1) peer-reviewed scientific publications receiving more attention in non-scientific media are more likely to be cited than scientific publications receiving less popular media attention, and (2) the non-scientific media is associated with the scientific agenda. These results may inform scientists who increasingly use popular media to inform the general public and scientists concerning their scientific work. These results might also inform administrators of higher education and research funding mechanisms, who base decisions partly on scientific impact.

## Introduction

Scientific research and the resulting peer-reviewed publications are highly valued in the evaluations of university faculty [1], as well as in the evaluations of a university’s commitment to research. Research productivity and impact are important factors in decisions that affect faculty hiring and pay, and allocation of ultra-competitive research funds [2–4]. Scientific impact (SI; herein defined generally as the amount of attention given to scientific research by scientists) is difficult to quantify, although SI is often thought to reflect research productivity and prestige. SI is often quantified via different citation counts; e.g., the number of times a scientific publication is cited in peer-reviewed literature, average number of citations for publications within a particular journal, or number of career citations for a specific researcher. Using citation counts to evaluate success of a given publication, journal, or researcher has its limitations because citation counts vary substantially between scientific disciplines [5] and researcher career stages [6], making it difficult to compare citation rates across disciplines and investigators. Additionally, a measurement of SI (as previously defined) using citation counts does not account for another important facet of scientific research: non-scientific impact (NSI; herein defined generally as the amount of attention given to scientific research by non-scientists in mainstream news outlets, online blogs, and/or social media). In some ways (as presently defined), NSI reflects the import of scientific research as perceived by members of the lay public. Scientists across various disciplines now increasingly use a wider variety of tools to disseminate scientific results to lay audiences using various media avenues [7]. Like SI, NSI is difficult to quantify. Further, the relationship between NSI and SI is unclear, although previous research has indicated a potential relationship between the mention of scientific research in popular media (e.g., mainstream news or social media) and scientific impact (e.g., citation count; [8–14]).

Mainstream media coverage of scientific research typically features scientific articles published in academic journals that fit the editorial focus and news values of mainstream media outlets [13, 15, 16]. Prior studies have described two different potential explanations for the relationship between NSI and SI: an earmark hypothesis and a publicity effect. The earmark hypothesis asserts that media outlets cover certain scientific studies because of their intrinsic value and, as a result, it predicts that the publicized studies would have garnered the same amount of SI without the benefit of media exposure [10, 11]. The publicity effect proposes that media coverage provides an SI boost for scientific studies that they would not have received on their own [10, 11, 17]. Based on this idea, academic publishers and authors have engaged in systematic public relations efforts to secure media coverage [12, 18]. In addition, public relations scholars have found empirical evidence that media coverage can influence perceptions and behavior of media consumers in a variety of different contexts [19–24]. While some researchers have focused upon news media coverage while studying the concept of publicity, other researchers have examined the relationship between social media and SI [12, 25, 26]. However, in the current digital media environment, information flows bidirectionally between traditional news outlets and social media platforms, making it difficult to study media sources in isolation [27, 28].

The purpose of this study was to investigate potential relationships between NSI (mainstream news and social media) and SI, with an emphasis on scientific research regarding physical health and exercise. More specifically, we investigated potential relationships between (1) popular (i.e., non-scientific) media attention given to scientific research regarding physical health and exercise, and (2) the attention given by scientists to the same research (*viz*, citations). We hypothesized that NSI and SI would be positively correlated; i.e., that scientists (like non-scientific consumers of popular news and social media) are more likely to think, study, and write about issues that either have received, or will yet receive, more attention in the mainstream news and social media. To account for several other factors that likely influence SI (in addition to NSI), we also attempted to quantify relationships between SI and scientific reputation of the scientist/author (AR) and academic journal (JR) in which the scientific research was published.

## Methods

To accomplish the purpose of this study, 818 peer-reviewed scientific articles describing original research studies were analyzed. All articles were chosen from a single discipline so that discipline-specific metrics associated with publication data (e.g., journal impact factors and author h-index values) were comparable across articles. The year of each publication was either 2007 or 2008. Each publication appeared in one of five prominent academic journals from the Sports Sciences subject category of the Web of Science (WOS; Clarivate Analytics, Philadelphia, PA, USA) scientific citation indexing service; these journals were chosen because we thought they provided a good representation of reputable physical health and exercise journals. Three of the journals (*Medicine and Science in Sports and Exercise*, *The American Journal of Sports Medicine*, and *Journal of Applied Physiology*) were first quartile journals in 2007 and 2008. One journal was second quartile in 2007 and 2008 (*European Journal of Applied Physiology*), while one was a third and first quartile journal in 2007 and 2008, respectively (*Journal of Science and Medicine in Sport*). All original research articles published in each journal during 2007 and 2008 were analyzed, except for *The Journal of Applied Physiology*, which publishes substantially more articles annually than the other journals; 200 articles were randomly sampled from this journal to prevent overrepresentation of this journal. All journal articles were accessed and associated journal article characteristics analyzed between October 2017 and May 2018.

The Altmetric Attention Score (AAS) was used as one of the measures representing NSI. The AAS is a weighted count of various non-scientific mentions of the scientific research/publication, including mainstream news, online blogs, Wikipedia, and Twitter and Facebook [29]. This approach was chosen because the AAS is a practical, accepted, single count of attention garnered across the presently large [30, 31], and still growing, spectrum of different forms of non-scientific media. The AAS was manually obtained for each original research article via the Altmetric bookmarklet, which was downloaded from the altmetric.com website; 74% of the original research articles had an Altmetric score of zero, indicating no measurable online attention. Because the AAS represents a diverse variety of non-scientific sources, it can be difficult to clearly understand the role of specific source types (e.g., social media versus newspaper mentions) within the AAS [32]. To better understand the specific influence of social media on NSI, separate from the influence of the AAS, we included social media mentions as an additional observed variable, in addition to the AAS. This variable, social media mentions, was represented by an aggregate of two of the most prominent social media platforms, Facebook and Twitter (e.g., 1 Facebook mention + 1 Twitter mention = 2 social media mentions). This information was gathered through the detailed AAS breakdown that is available for each article via the Altmetric bookmarklet.

Citation counts and several other article usage metrics were used to represent SI for each publication. Citation counts reported by both Scopus (Elsevier, Amsterdam, Netherlands) and WOS were used. Journal impact factor and subject category (Sport Sciences) rank were collected via the InCites Journal Citation reports in WOS. The field-weighted citation impact score from Scopus was also used (Orthopedics and Sports Medicine subject area). Journal impact factor, WOS subject category rank, and field-weighted citation count each represent methods designed to quantify journal quality and scientific impact; each method attempts to account for differences across scientific disciplines and publication dates. The Sports Sciences subject category (WOS) is smaller (currently 83 journals) than the Orthopedics and Sports Medicine subject area (Scopus; currently 244 journals). Additionally, counts of individual article abstract views and readers on the reference managing site Mendeley (Elsevier, Amsterdam, Netherlands) were used to help represent SI. These Mendeley values were extracted from the Mendeley website manually using a title search for each analyzed article. In almost every case, searching via the article title yielded the correct article; however, in a few cases, it was necessary to search via the lead author name which yielded the correct result. Whether by title or author search, only one corresponding article was found and recorded for each inquiry. Initially, we intended to collect Mendeley readership data using the Altmetric bookmarklet, but Mendeley readership data were not available for all articles using the bookmarklet.

Because AR and JR likely affect citation counts [33, 34], we attempted to account for these factors in the current analysis. AR was evaluated using the WOS h-index for the lead and corresponding authors. Reputation for the lead and corresponding authors’ institution(s) were also used to help represent AR, and these were evaluated based upon research output rankings for the academic institution(s) of the lead and corresponding author at the time of publication; the Academic Ranking of World Universities (Shanghai Ranking) was used to evaluate institutional ranking, which is reputable and publicly available. JR was evaluated via the ranking of the journal within the WOS Sports Science subject category, as well as 1- and 5-year journal impact factors and journal impact factor percentiles (all collected via WOS).

After removing any publication with partially-missing data, 801 of the 818 aforementioned scientific publications remained for analysis. A sample size of 801 with 32 parameters results in a sample-to-parameter ratio of approximately 25, which is larger than necessary for estimation and evaluation of model fit [35, 36]. To ensure that observations were optimally suited for structural equation modeling, skewed variables were transformed. A Box-Cox approach was used to find the optimal transformation toward normality [37]; to avoid computational problems associated with log(0), we used the log(x+1) transformation whenever the Box-Cox procedure selected the log transformation as optimal. Additional details concerning the transformation selected for each variable are presented in Table 1. The structural equation modeling approach was used to consider the relationships among four latent constructs (factors): AR (*f*_AR_), JR (*f*_JR_), NSI (*f*_NSI_), and SI (*f*_SI_). Structural equation modeling is an oft used tool for characterizing complex interrelationships among variables within a network of observed or latent variables [38, 39]. In order to identify an association between *f*_NSI_ and *f*_SI_, while accounting for potential influence of the *f*_AR_ and *f*_JR_, we chose to model the effects of the first three factors (*f*_AR_, *f*_JR_, and *f*_NSI_) on the last factor (*f*_SI_). As described in the Results section, we found that a latent variable model was a generally illuminating approach, succinctly characterizing the relationships between *f*_SI_ and each of the other factors (*f*_AR_, *f*_JR_, and *f*_NSI_).

**Table 1:** Table of descriptive statistics for transformed variables included in the final model. For each of the 13 variables, the article count, average, standard deviation (SD), median, interquartile range (IQR), and minimum and maximum values are presented. All descriptive statistics are calculated for all journals and within the distinct journal quartiles. As many variables have been transformed, a description of the transformation performed (if any) is also included.

Variable	Quartile 1	Quartile 2	Quartile 3	Overall
**Lead Author H-index**				
Transformation, if any				sqrt(Lead Author H-index)
Count	507	172	123	801
Mean (SD)	3.33 (1.42)	3.64 (1.41)	2.96 (1.46)	3.34 (1.43)
Median (IQR)	3.16 (1.79)	3.61 (1.74)	2.83 (1.87)	3.16 (1.79)
Min, Max	0.00, 10.20	1.00, 7.35	1.00, 7.00	0.00, 10.20
**Institution Ranking**				
Transformation, if any				log(Institution Ranking + 1)
Count	507	172	123	801
Mean (SD)	5.45 (1.38)	5.92 (1.15)	6.01 (1.07)	5.64 (1.31)
Median (IQR)	5.53 (2.40)	6.11 (1.74)	6.91 (1.99)	5.86 (2.07)
Min, Max	0.69, 6.91	2.64, 6.91	3.74, 6.91	0.69, 6.91
**Corresponding Author H-index**				
Transformation, if any				sqrt(Corresponding H-index)
Count	507	172	123	801
Mean (SD)	3.91 (1.48)	4.22 (1.51)	3.31 (1.56)	3.89 (1.52)
Median (IQR)	3.74 (2.02)	4.12 (2.15)	3.16 (1.89)	3.74 (2.17)
Min, Max	1.00, 10.20	1.00, 7.87	1.00, 7.00	1.00, 10.20
**Scopus Citation Count**				
Transformation, if any				sqrt(Scopus)
Count	507	172	123	801
Mean (SD)	6.01 (2.82)	4.96 (2.22)	4.68 (2.21)	5.58 (2.68)
Median (IQR)	5.48 (3.38)	4.58 (3.22)	4.47 (2.22)	5.10 (3.26)
Min, Max	1.00, 20.88	0.00, 12.25	0.00, 12.29	0.00, 20.88
**Web of Science Count**				
Transformation, if any				sqrt(Scopus)
Count	507	172	123	801
Mean (SD)	5.77 (2.68)	4.79 (2.16)	4.29 (2.13)	5.34 (2.56)
Median (IQR)	5.20 (3.20)	4.53 (3.10)	4.12 (1.97)	4.90 (3.24)
Min, Max	0.00, 20.27	0.00, 11.27	0.00, 11.45	0.00, 20.27
**Field Weighted Citation Count**				
Transformation, if any				sqrt(WoS)
Count	507	172	123	801
Mean (SD)	0.93 (0.53)	0.70 (0.38)	0.70 (0.42)	0.85 (0.50)
Median (IQR)	0.90 (0.70)	0.64 (0.55)	0.62 (0.52)	0.78 (0.61)
Min, Max	0.00, 3.47	0.00, 1.68	0.00, 1.99	0.00, 3.47
**Abstract Views**				
Transformation, if any				(AbsViews)^0.30
Count	507	172	123	801
Mean (SD)	6.78 (2.60)	6.12 (1.42)	7.30 (1.70)	6.72 (2.30)
Median (IQR)	6.92 (3.24)	6.10 (1.80)	7.14 (1.76)	6.72 (2.84)
Min, Max	1.00, 15.80	2.91, 10.97	0.00, 11.37	0.00, 15.80
**Mendeley Readership**				
Transformation, if any				(Mendeley)^0.40
Count	507	172	123	801
Mean (SD)	4.05 (1.72)	3.64 (1.54)	4.64 (1.66)	4.05 (1.70)
Median (IQR)	3.85 (1.94)	3.57 (1.84)	4.52 (1.59)	3.95 (1.90)
Min, Max	0.00, 12.25	1.00, 8.51	0.00, 10.35	0.00, 12.25
**Journal Impact Factor %**				
Transformation, if any				None used
Count	507	172	123	801
Mean (SD)	96.61 (2.38)	81.25 (0.00)	66.76 (15.26)	88.76 (12.80)
Median (IQR)	96.85 (2.85)	81.25 (0.00)	78.17 (31.64)	95.07 (15.60)
Min, Max	92.36, 100.00	81.25, 81.25	46.53, 78.17	46.53, 100.00
**1-year Impact Factor**				
Transformation, if any				None used
Count	507	172	123	801
Mean (SD)	3.59 (0.38)	1.75 (0.00)	1.70 (0.36)	2.91 (0.96)
Median (IQR)	3.63 (0.57)	1.75 (0.00)	1.91 (0.82)	3.40 (1.88)
Min, Max	2.86, 3.97	1.75, 1.75	1.09, 1.91	1.09, 3.97
**5-year Impact Factor**				
Transformation, if any				None used
Count	507	172	123	801
Mean (SD)	3.77 (0.15)	2.10 (0.00)	1.93 (0.20)	3.13 (0.85)
Median (IQR)	3.69 (0.18)	2.10 (0.00)	2.05 (0.44)	3.64 (1.72)
Min, Max	3.64, 4.08	2.10, 2.10	1.61, 2.05	1.61, 4.08
**Altmetric Score**				
Transformation, if any				log(Altmetric + 1)^0.7
Count	507	172	123	801
Mean (SD)	0.36 (0.67)	0.26 (0.56)	0.38 (0.70)	0.34 (0.65)
Median (IQR)	0.00 (0.77)	0.00 (0.00)	0.00 (0.77)	0.00 (0.77)
Min, Max	0.00, 3.12	0.00, 3.08	0.00, 2.67	0.00, 3.12
**Social Media Mentions**				
Transformation, if any				log(SocMedia + 1)
Count	507	172	123	801
Mean (SD)	0.34 (0.80)	0.20 (0.61)	0.25 (0.62)	0.30 (0.74)
Median (IQR)	0.00 (0.00)	0.00 (0.00)	0.00 (0.00)	0.00 (0.00)
Min, Max	0.00, 4.64	0.00, 5.07	0.00, 2.83	0.00, 5.07

**Fig 1:**
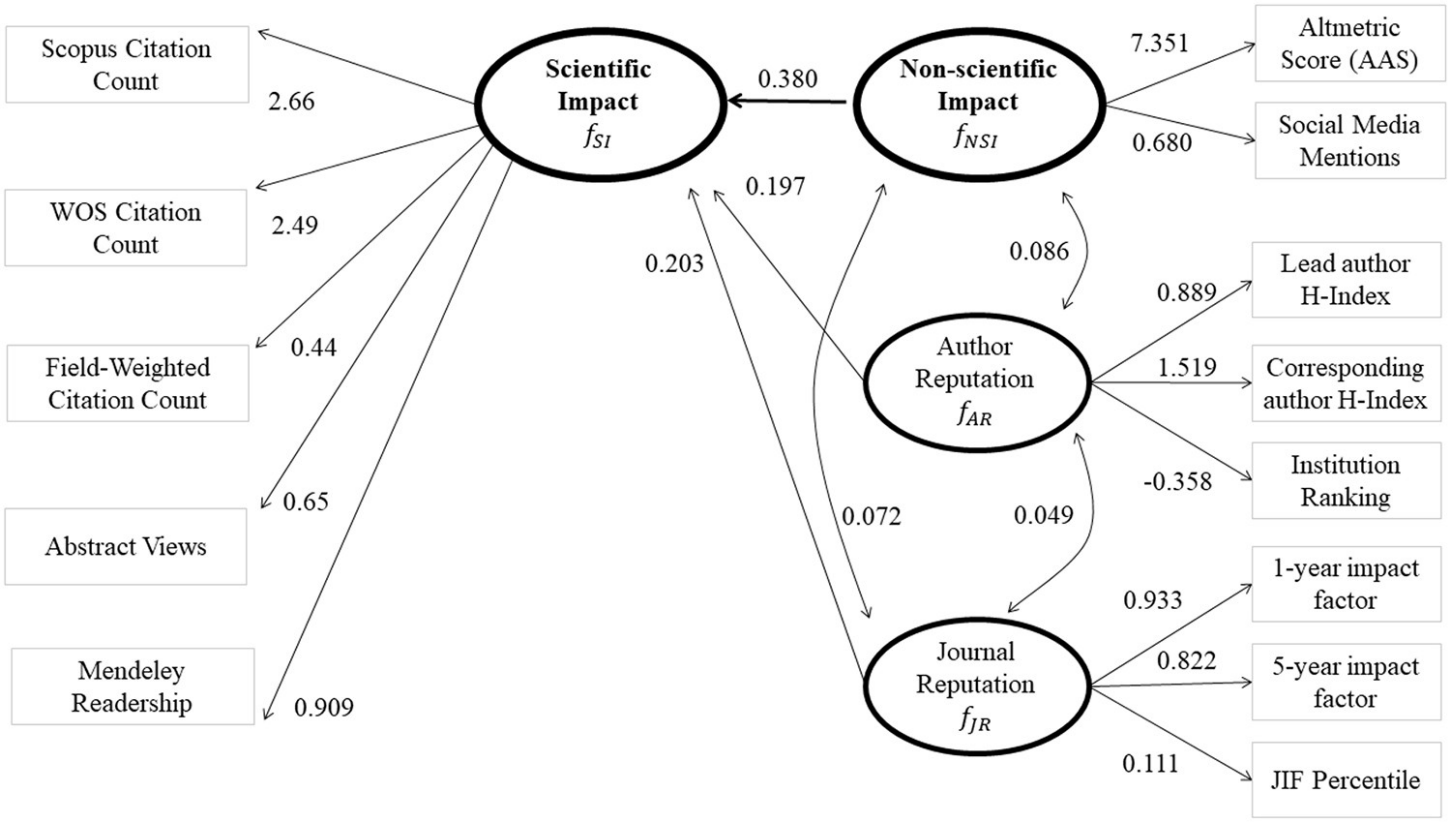
A graphical representation of the structural equation model used in the present study to investigate a potential relationship between attention given to scientific research in non-scientific media and the peer-reviewed scientific literature. Unidirectional relationships are denoted by single headed arrows. Covariances between two factors are shown using double headed arrows. Factors are denoted by bolded oval shapes and observed variables are denoted by rectangles. The model indicates that there is a strong relationship between attention given in non-scientific media (*f*_NSI_), by the general public, and scientific literature (*f*_SI_), by scientists.

The structural equation model is shown graphically in Fig 1. The latent factor *f*_*AR*_, is associated with institution ranking, as well as the lead and corresponding authors’ h-indices. The second latent factor (*f*_*JR*_) is the driving factor behind the observed variables of the 1- and 5-year impact factors for the journal, as well as the percentile for the journal impact factor within the exercise and wellness area (a subject category created by WOS). The third latent factor (*f*_*NSI*_) is related to AAS and social media mentions, as previously described. Finally, the factor for SI (*f*_*SI*_) is associated with the following observable variables: both Scopus and WOS citation counts, Field Weighted Citation Count, as well as counts of individual article abstract views and readers on the reference managing site Mendeley. For each variation of the structural equation model that we fit, we first considered goodness-of-fit indices to assess overall model fit including the *χ*^2^ goodness-of-fit test, root mean square error of approximation (RMSEA) [40], standardized root mean square residual (SRMR) [41], and Bentler’s comparative fit index (CFI) [42]. When using the *χ*^2^ test statistic to assess model fit, the probability of rejecting the proposed model increases with the number of observations, rendering the undesirable property of increasing the chance of rejecting models with reasonable fit as the sample size increases [43]. Consequently, researchers often rely on goodness-of-fit measures such as RMSEA, SRMR, and CFI to assess model adequacy. The RMSEA metric is calculated using the *χ*^2^ test statistic and quantifies the excess lack of fit for the model. One recommended criterion is that an RMSEA value less than 0.06 represents a good fit [44]. Other researchers have suggested 0.01, 0.05, and 0.08 to indicate excellent, good, and mediocre fit, respectively [45]. The SRMR is a second absolute measure of fit that quantifies the difference between the model-predicted covariance matrix for the variables and the sample covariance matrix. An SRMR value of zero indicates a perfect fit and a value of one indicates the opposite. Hu and Bentler suggest that an SRMR value that is less than 0.08 indicates a good fit [46]. Finally, CFI quantifies the improvement in the *χ*^2^ test statistic when comparing to a baseline model consisting of uncorrelated variables. CFI ranges from zero to one, with a value of one indicating that the chosen model has removed all of the lack of fit associated with the baseline model. Hu and Bentler recommend that CFI values greater than 0.95 indicate a good fit [46].

## Results and discussion

A structural equation model was produced to investigate interrelationships among measurable (i.e., observed) variables associated with NSI and SI, and AR and JR (Fig 1). As was described in more detail in the Methods section, the adequacy of the model was assessed using a suite of goodness-of-fit statistics and corresponding accepted rules of thumb. A general consensus among the following assessments indicated that the model reasonably characterizes relationships among the measurable variables associated with NSI, SI, AR, and JR: RMSEA, SRMR, and Bentler’s CFI (CFI; *χ*^2^ = 453.796, with p value < 0.0001; RMSEA = 0.091; SRMR = 0.057; CFI = 0.951). We considered the three goodness-of-fit metrics of CFI, RMSEA, and SRMR together as a holistic assessment of model fit; because two of the three metrics met our criterion for good fit, we determined that our model was appropriate and adequately fit the data. Descriptive statistics for all involved variables are presented in Table 1.

The aforementioned structural equation model was fit in Mplus software [39] (Muthén & Muthén, Los Angeles, CA, USA). Standardized coefficients are reported herein for ease of interpretability. A path diagram for the model is presented in Fig 1. Important predictive relationships exist (all *p* values were < 0.001) between each of the exogenous factors (*f*_*AR*_, *f*_*JR*_, and *f*_*NSI*_) and the SI factor (*f*_*SI*_). Within Fig 1, unidirectional impacts are indicated by the arrows pointing from *f*_*AR*_, *f*_*JR*_, and *f*_*NSI*_, toward *f*_*SI*_, and also by the arrows relating these factors with their associated observed variables. The curved bi-directional arrows in Fig 1 represent covariances between factors that do not have a direct relationship with each other. The standardized factor loading estimate for NSI onto SI was approximately 0.380, which is nearly twice as large as the standardized factor loading estimate for either of the other two exogenous factors (f_AR_ and f_JR_) on SI (0.197 and 0.203, respectively). We interpret these standardized factor loading estimates to indicate that for a scientific publication, a one standard deviation increase in NSI is associated with a 0.380 standard deviation increase in SI for that same publication, holding all other considered factors constant. Similarly, a one standard deviation increase in AR would correspond to a 0.197 standard deviation increase in SI, and a one standard deviation increase in JR would correspond to a 0.203 standard deviation increase in SI, holding all other considered factors constant. By comparing the differences in magnitude between these standardized factor loading estimates, we conclude that NSI has a strong association with SI of an article. Importantly, however, we note that the *R*^2^ value for *f*_*SI*_ is 25.2%, meaning that only 25.2% of the variability in SI can be explained by the combination of NSI, AR, and JR.

A strong relationship was observed between each defined variable (shown in the rectangles in Fig 1) and its associated factor (shown in the ovals in Fig 1). Additionally, all factor loadings exhibited the expected direction: all were positive except for Institutional Ranking, which uses smaller values to represent institutions with better reputations. Each of these loadings had an associated *p* value of less than 0.001. The relative size of the factor loadings associated with a given factor indicates the relative importance of each observed variable in the definition of the factor. For example, the SI factor loads onto the Scopus and WOS (Clarivate Analytics, Philadelphia, PA, USA) citation count variables most heavily, indicating how each factor is quantifying the notion of SI. Similarly, corresponding author h-index is the most important observed variable in defining *f*_*AR*_, one- and five-year impact factors are the most important components of *f*_*JR*_, and the AAS is most important in defining *f*_*NSI*_. Fig 1 also indicates weak positive correlations among the three exogenous factors: *corr* (*f*_*AR*_, *f*_*JR*_) = 0.049 (*p* = 0.167), *corr* (*f*_*AR*_, *f*_*NSI*_) = 0.086 (*p* = 0.023), and *corr* (*f*_*JR*_, *f*_*NSI*_) = 0.072 (*p* = 0.063).

In review, the purpose of this study was to determine whether non-scientific attention given to scientific research in various forms of popular media (NSI) is related to the amount of attention paid to the same research by fellow scientists, via scientific citations (SI), while accounting for some of the other factors also thought to influence SI. The present results revealed a strong positive association between NSI and SI, indicating that scientific experiments receiving more attention in non-scientific media such as mainstream news and/or social media are cited more in the peer-reviewed scientific literature. The present results demonstrate that news outlets and social media are either discussing the most scientifically impactful papers, or that increased coverage of a scientific article increases the likelihood of an article receiving scientific citations. It was also determined that author and journal reputations are valuable predictors of SI, but the effect size for NSI is roughly equivalent to the combined effect size for the two reputation factors (AR and JR). While the current model looks foremost at unidirectional effects of AR, JR, and NSI, on SI, we cannot confidently assert that these effects are causal, and we cannot speak to the chronology of the present effects which is also a shortcoming of other related previous research [10, 11, 14, 17, 47]. Specifically, the current results do not indicate that scientists, or their associated institutions, will experience greater scientific impact by enlarging their media relations staff or expanding their social media outreach. On the other hand, given the strong association between NSI and SI, this research does indicate that scientists and institutions should carefully consider the impact of popular and social media when striving to expand their influence or evaluate the influence of individual scientists. Among other benefits, a clearer understanding of the association between NSI and SI might assist scientists in effectively connecting the general public to the most impactful research.

**Fig 2:**
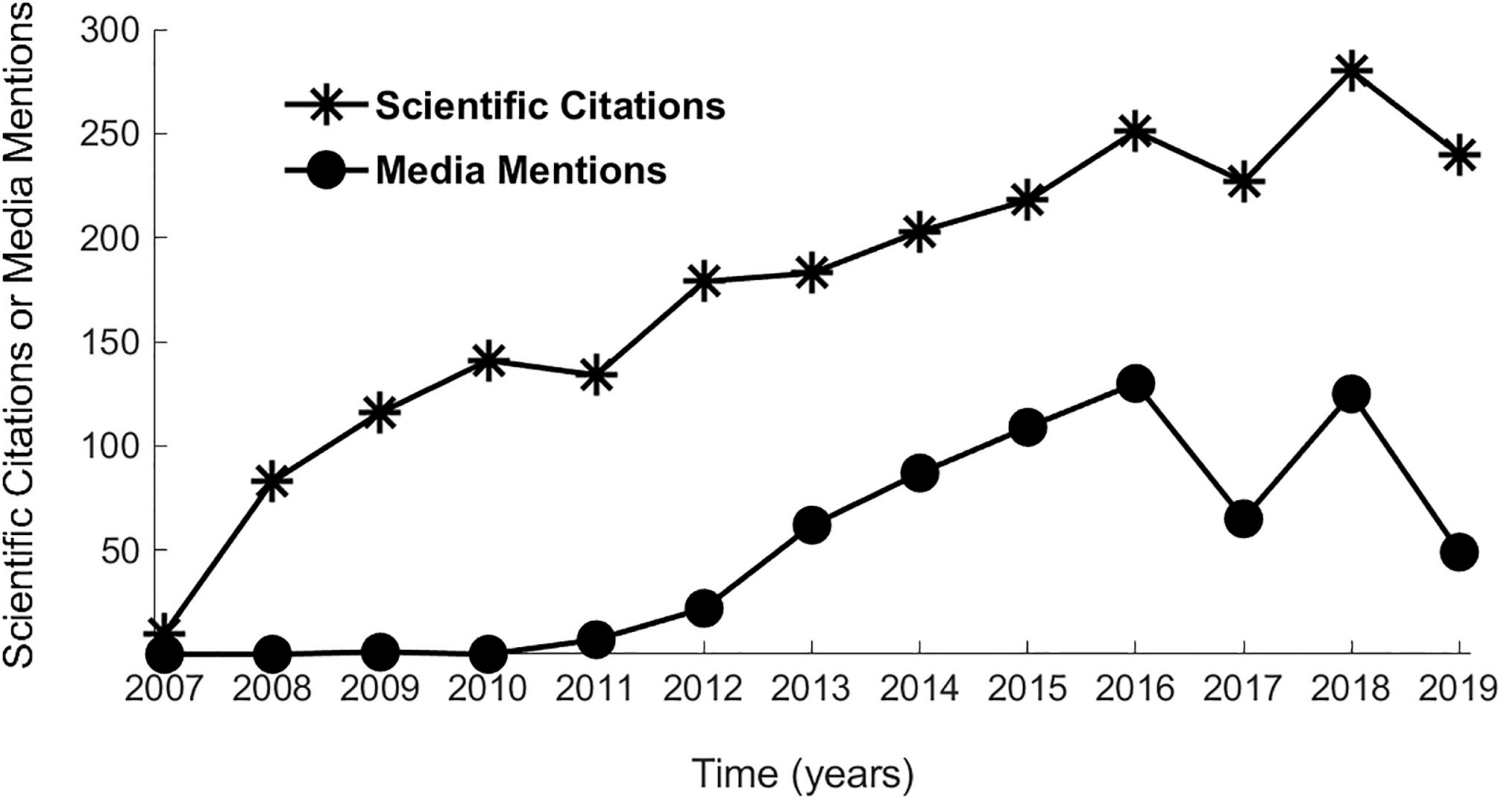
Results from the post hoc sub-analysis examining a potential temporal sequence of distinct popular media mentions and scientific citations for 18 peer-reviewed scientific publications describing original research concerning physical health and exercise. The observation that the two lines are somewhat parallel indicates no consistent temporal sequence for mentions in popular media and scientific citations; i.e., it is unclear which generally come first (popular media mentions or scientific citations).

Again, it is important to not conclude that the present results support a particular temporal sequence of NSI and SI, as has been implied by some previous researchers [10, 11, 17] who discussed a publicity effect of non-scientific media on SI (i.e., non-scientific media boosting subsequent SI). The present model (Fig 1) does not indicate causality and temporal sequence should not be assumed. We conducted a post hoc sub-analysis, as a limited test of temporal sequence, by analyzing dates of the identified non-scientific mentions and scientific citations for 18 of the 801 present scientific publications (six publications from each of the first, second, and third quartiles of the Sports Sciences WOS subject category rank). The results of this sub-analysis are presented in Fig 2. Generally, the parallel nature of the lines representing NSI and SI (Fig 2) suggest that non-scientific mentions and scientific impact might occur simultaneously, contradicting the ideas that NSI consistently provides a subsequent boost to SI, or vice versa. Although interesting, the results of this sub-analysis represent only a small sample and are otherwise limited. For example, only aggregated data were considered. Scientific citations and media mentions were not linked for individual publications, so the parallel nature of the two lines in Fig 2 might be due to factors not related to a temporal relationship between scientific citations and media mentions. Larger and more comprehensive studies are needed to clearly understand the temporal sequence of non-scientific mentions and scientific impact for research concerning physical health and exercise, and other disciplines.

It is important for most scientific research to be generally accessible to a non-specialist audience. Future research should explore potential news values (e.g., significance, prominence, proximity, etc.) inherent in scholarly research that may motivate the news media and science-centric social media accounts to report on some studies while ignoring others (i.e., the earmark hypothesis). While acknowledging that not all important research will be “newsworthy,” as defined by traditional news values, a clear understanding of how producers of popular media select topics to talk and write about can help researchers learn to frame their research for consumption by members of the general society. Future research may assist scientists in knowing how to present their research in palatable ways in mainstream news and social media, making their research both more accessible to the general public as well as actionable for industries associated with the research discipline. Future related research might also help scientists better understand specific motivations and purposes for different users of social media who mention scientific research, as it is known that different social media platforms are sometimes used for different purposes (e.g., societal impact, visibility, or education [32]). Although limited in some ways, companies such as Kudos Innovations [48] now offer various ways for scientists to present their research in ways that are more friendly to the lay population.

The present results corroborate the established notion that quantification of SI is difficult. SI is complicated, with multiple dimensions, and the present finite representations of SI do not comprehensively represent this complex concept. This is substantiated by the present finding that the current model (involving NSI, AR, and JR) predicted only slightly more than a quarter of the variance in SI. Despite the complexity and potential misunderstanding of SI, it is commonly and perhaps over-confidently used to inform important decisions in academia, like decisions concerning research funding allocations, and faculty hiring and promotions. Numerous factors influence SI [49], many of which were not considered in the present analysis. Some of these other factors include but are not limited to article type (e.g., review article, short note, or book chapter) [50], publication language [51], number of listed coauthors [52, 53], and availability of the publication (i.e., open access or traditional subscription-based journals) [54]. Even technical problems like incorrect citations within articles [55, 56] are (unfortunately) quite common and influence citation rates; one noteworthy study in this area reported that nearly half of all citations in a sample of scientific articles were incorrect [55].

Although research concerning physical health and exercise is not the primary focus of this paper, the context of physical health and exercise facilitates some valuable discussion. Physical health and exercise are topics that receive vast attention from non-scientific sources, and the appropriateness of some of this attention may be dubious. Similar to research in other disciplines, it is important that, generally speaking, the most meaningful matters of physical health and exercise receive the most attention in non-scientific media; NSI, such as social media and other private media outlets, influences people’s choices related to health. If a research article is having a large impact on society, it probably deserves further attention from researchers. The present findings are important because there is a dearth of research concerning the relationship between SI and NSI in physical health and exercise (although we hypothesize that present research applies to other scientific disciplines). Physical health and exercise are often mentioned in popular media, including mainstream news and social media, and it is not unusual for results from scientific research to be included in these mentions. To ensure the health and safety of the general public, it is imperative to know in what ways popular media impact and scientific impact are related (if at all) for scientific research concerning physical health and exercise. If NSI and SI are unrelated for research concerning physical health and exercise, then there is reason for concern that society consumes research that is not the most important, but merely the most interesting. The idea that the general public is being offered, for consumption, research of less impact, relative to more impactful research is concerning. An example of this, from the physical health and exercise literature, is a study [57] that received immense media attention (an AAS of 729—top 5% of all research output scored by Altmetric) that was arguably undeserved: the study was a pilot study, and was explicitly described as such. While the result was intriguing, the study involved only six young healthy subjects and was not representative of a large part of the general adult American population.

In addition to the limiting factors already discussed, other limitations exist for the present study. Although we attempted to control for all variables that likely influence NSI and SI, some confounding variables were not completely controlled for, including scientific merit of and popular interest of the observed original research articles. These two confounding variables were considered at the outset of this research, yet completely accounting for these two variables was beyond our means. The establishment of expert committees to evaluate scientific merit and popular interest of each original research study presently analyzed was considered, but the cost of such committees was beyond our budgetary restraints and inherently subjective. Another factor that likely limits current relevance of the present results is our decision to analyze research articles only from the years 2007–08. Social media use has clearly increased since 2007–2008, and current relationships between NSI and SI are likely different. Major social media platforms (e.g., Facebook) were not as prominent in 2007–2008 as they are today, and research performed in 2007–2008 is less likely to appear on social media today, relative to more recent research [58]. We opted to choose two years (2007–2008) in the recent past that optimized popular media coverage on the internet while also allowing time for some potential scientific citations to accrue, because some researchers have described a risk in citation related research of extracting data before sufficient time has been given for scientific citations to accrue [58]. This all had to be balanced with an ability to find news media mentions on the internet. To find this coverage, we chose to use data collected by the Altmetric organization, which also presents some limitations. Altmetric did not begin collecting news media coverage for research articles until October 2011 (altmetric.com); thus coverage of research articles deleted by popular news sources prior to October 2011 was not included in our evaluation. Because media sources are most likely to report on recently published research articles [59, 60] it is possible that the number of missed sources was not insignificant, and we do not know the number of undetectable media mentions. AAS is not a comprehensive measure and is limited in other ways. For example, approximately three quarters of the articles presently analyzed had AAS’s that equaled zero, while only four of the analyzed articles had Mendeley readership values of zero. Interestingly, related to Mendeley readership, the presently analyzed articles exhibited relatively good coverage: more than 99% of the articles had a non-zero Mendeley readership value, relative to previously reported averages that ranged from 82% in 2013 to 89% in 2009 [61]. This good coverage existed despite the fact that only one of the five presently analyzed journals belongs to Elsevier (Mendeley was acquired by Elsevier in 2013). Another limitation associated with the use of AAS is due to the heterogeneous nature of the AAS, which limits the ability to understand specific constituents; research aiming to more clearly understand more precise contributions to the AAS obviously is needed and requires additional resources. Another final limitation discussed herein relates to the fact that research articles for this study focused upon the discipline of physical health and exercise. The nature of relationships among media attention, author reputation, journal reputation, and scientific impact may prove to differ across disciplines; however, our study argues that intended and unintended impacts of such relationships should at least be acknowledged when evaluating the scientific impact of articles or authors associated with academic publishing.

## Conclusions

In summary, the current results reveal a strong relationship between the (1) amount of attention peer-reviewed scientific research concerning physical health and activity receives through popular media and (2) amount of attention the same research receives from fellow scientists, reflected by number of citations in peer-reviewed scientific literature. The direction of this association, however, cannot be ascertained via the current results; the popular media might capably perceive the most impactful scientific research to report upon, or the popular media attention might cause an increase in subsequent scientific impact. These findings have several potential applications outlined herein. Much remains to be discovered concerning interactions between popular media, generally produced by and for non-scientists, and the scientific literature which has historically been written by and for the scientific community.
